# Circulating Interleukin-7 in Human Pulmonary Arterial Hypertension

**DOI:** 10.3389/fcvm.2021.794549

**Published:** 2021-12-08

**Authors:** Franziska Diekmann, Ekaterina Legchenko, Philippe Chouvarine, Ralf Lichtinghagen, Harald Bertram, Christoph M. Happel, Georg Hansmann

**Affiliations:** ^1^Department of Pediatric Cardiology and Critical Care, Hannover Medical School, Hanover, Germany; ^2^Institute of Clinical Chemistry, Hannover Medical School, Hanover, Germany

**Keywords:** IL-7, VEGF-C, ICAM-1, VCAM-1, innate and adaptive immunity, pulmonary arterial hypertension, transpulmonary pressure gradient, vascular injury

## Abstract

**Objectives:** Interleukin-7 (IL-7) secures B cell maturation, regulatory T and natural killer (NK) cell survival, and homeostasis, all of which are important for beneficial immunomodulation in pulmonary arterial hypertension (PAH). However, the role and potential impact of IL-7, VEGF-C and the vascular injury markers ICAM-1, and VCAM-1 on the pathobiology and severity of PAH is unknown.

**Methods:** EDTA blood was collected during cardiac catheterization from the superior vena cava (SVC), pulmonary artery (PA), and ascending aorta (AAO) in children with pulmonary hypertension (PH) [*n* = 10; 9.1 (3.9–18.5) years] and non-PH controls [*n* = 10; 10.5 (2.0–17.3) years]. Compartment-specific plasma concentrations of IL-7, VEGF-C, aldosterone, ICAM-1, and VCAM-1 were determined using Meso Scale Discovery's multi array technology and the LIAISON Aldosterone Assay.

**Results:** Children with PH had approximately 50% lower IL-7 (*p* < 0.01) and 59% lower VEGF-C plasma levels (*p* < 0.001) in the SVC, PA, and AAO versus non-PH controls. IL-7 and VEGF-C concentrations negatively correlated with the pulmonary vascular resistance (PVR)/systemic vascular resistance (SVR) ratio (rho = −0.51 and *r* = −0.62, respectively). Central-venous IL-7 strongly positively correlated with VEGF-C (*r* = 0.81). Most patients had a step down in ICAM-1 and VCAM-1 plasma concentrations across the pulmonary circulation and both ICAM-1 and VCAM-1 transpulmonary gradients negatively correlated with invasive hemodynamics.

**Conclusion:** This manuscript is the first report on decreased circulating IL-7 and VEGF-C plasma concentrations in human PAH and their inverse correlations with invasive surrogates of PAH severity. Additional and larger studies are needed to explore the role of the immune-modulatory IL-7 and VEGF-C in pediatric and adult PAH.

## Introduction

The inflammatory response and its resolution, delivered by the innate and adaptive immune systems, are important for development, disease severity, and responses to therapy, in many patients with pulmonary arterial hypertension (PAH) (1, 2). The pro-inflammatory phenotype of pulmonary endothelial cells in PAH is characterized by increased surface expression of intercellular adhesion molecule 1 (ICAM-1), vascular cell adhesion molecule 1 (VCAM-1), and E-selectin, and accompanied by an excessive release of cytokines and chemokines (3). The sequelae include impairment of angiogenesis and cardiovascular repair mechanisms, and promotion of pulmonary vascular remodeling. Emerging evidence suggests that natural killer (NK) cells (4–6) and regulatory T cells (Tregs, CD4+) (7) play major, protective immune-modulatory roles in both preclinical and human PAH, and associated right ventricular dysfunction (RVD). Additional protective factors include hepatocyte growth factor (HGF) that reduces PAH severity and inflammation by attenuation of NF-kB signaling (8), and suppresses vascular medial hyperplasia and extracellular matrix accumulation (9) in the monocrotaline PAH rat model. Interleukin-7 (IL-7) forms a heterodimer with HGF and secures B cell maturation, T and NK cell survival, and homeostasis (10). However, in contrast to HGF, the role and potential impact of IL-7 on the pathobiology and severity of PAH is unknown. In addition, VEGF-C, aldosterone, and the aforementioned vascular injury markers ICAM-1 and VCAM-1 have not been studied in children with PAH, and have not been correlated with invasive hemodynamics.

Here, we report on the compartment-specific plasma concentrations of IL-7, VEGF-C, aldosterone, ICAM-1 and VCAM-1 in the superior vena cava (SVC), pulmonary artery (PA), and ascending aorta (AAO). We investigated 10 well-phenotyped children with moderate PH (WHO functional class 2–3) and 10 age-matched, non-healthy controls with left ventricular outflow tract obstruction (LVOTO; *n* = 9) and s/p reconstruction of a double aortic arch (*n* = 1), as previously described (11–13).

## Methods

A detailed description of the methods, including cardiac catheterization, simultaneous-compartmental blood draws, and enzyme-linked immunosorbent assays (ELISA) on EDTA plasma, can be found below:

### Study Population

10 PH patients and 10 non-PH controls underwent combined right and left heart catheterization. Non-PH controls had left ventricular outflow tract obstruction (LVOTO, *n* = 9) or s/p reconstruction of a double aortic arch (*n* = 1). EDTA blood samples, pressure recordings, and blood gas analysis (SpO2) were obtained near-simultaneously at three sites: superior vena cava (SVC), pulmonary artery (PA), and ascending aorta (AAO). To ensure compartment-specific analysis, none of the PH patients, and non-PH controls had intra- or extracardiac shunts. The legal caregivers of each study subject gave written informed consent. Inclusion criteria were defined according to the World Symposium on Pulmonary Hypertension in Nice (2018): mean pulmonary artery pressure (mPAP) > 20 mmHg (14, 15). Detailed information on PH-patients and non-PH controls including demographic data, functional status, and hemodynamics can be found in Table 1. The study has been approved by Hannover Medical School (#2200).

**Table 1:** Characteristics of PH patients and non-PH controls.

	**PH patients (*N* = 10)**	**non-PH controls (*N* = 10)**	***p*** **-value**
**Demographics**			
Age–years	9.1 (3.9–18.5)	10.5 (2.0–17.3)	n.s. (0.3822)
Male sex–*n* (%)	4 (40%)	6 (60%)	
Height – m	1.30 ± 0.09	1.39 ± 0.09	n.s. (0.3046)
Weight – kg	30.2 ± 7.2	33.9 ± 4.6	n.s. (0.2713)
BSA – m^2^	1.0 ± 0.1	1.1 ± 0.1	n.s. (0.3061)
**Clinical diagnosis**			
	1.1 IPAH−3 1.2 HPAH−3 1.4.3 Portal hypertension−1 1.4.4 PAH-repaired CHD−1 PH group 3 (lung disease)−2	LVOTO−9 s/p double aortic arch −1	
**Functional status**			
WHO FC	2.5 ± 0.2	1.0	
6 MWD–m, *n* = 5	344.0 ± 77.6	N/A	
NTproBNP (SVC)–ng/l	168.8 ± 50.8	79.6 ± 12.4	n.s. (0.1594)
**Key hemodynamics**			
**Cardiac catheterization**			
mRAP–mm Hg	3.7 ± 0.8	3.0 ± 0.7	n.s. (0.4585)
RVSP–mmHg	79.9 ± 10.2	25.6 ± 2.0	<0.0001
RVEDP–mm Hg	7.6 ± 1.1	4.4 ± 1.0	n.s. (0.0604)
sPAP–mm Hg	81.8 ± 10.9	22.4 ± 2.1	<0.0001
dPAP–mm Hg	35.9 ± 6.5	6.9 ± 1.5	0.0013
mPAP–mm Hg	56.9 ± 7.1	14.1 ± 1.5	<0.0001
mPAP/mSAP	0.8 ± 0.1	0.2 ± 0.03	<0.0001
PAWP–mm Hg	7.6 ± 1.5	8.0 ± 1.4	n.s. (0.8890)
mTPG–mm Hg	50.7 ± 6.9	6.0 ± 0.7	<0.0001
dTPG–mm Hg	30.6 ± 5.9	0.6 ± 0.3	0.0003
PVRi–WU·m^2^	13.3 ± 2.3	1.6 ± 0.2	<0.0001
SVRi–WU·m^2^	17.7 ± 2.0	16.1 ± 1.4	n.s. (0.7394)
PVR/SVR	0.7 ± 0.1	0.1 ± 0.01	<0.0001
Qpi–L/min/m^2^	4.0 ± 0.2	4.0 ± 0.1	n.s. (0.9281)
Qsi–L/min/m^2^	4.2 ± 0.3	4.3 ± 0.2	n.s. (0.6706)
Qp/Qs	1.0 ± 0.03	0.94 ± 0.03	n.s. (0.3033)
**Echocardiography**			
RVAWD–cm, PSAX, n = 8	0.7 ± 0.1	0.3 ± 0.03	0.0003
RVEDD–cm, PSAX, n = 7	2.2 ± 0.3	1.2 ± 0.1	0.0122
RV/LV endsys. ratio, PSAX	1.2 ± 0.1	0.5 ± 0.05	<0.0001
LV ecc. index (PSAX)	1.3 ± 0.1	0.9 ± 0.03	0.0005
S/D ratio, TRV jet, n = 8	1.5 ± 0.2	N/A	
TAPSE–cm, apical	1.7 ± 0.1	2.0 ± 0.08	n.s. (0.0847)
PAAT–ms, PSAX	85.8 ± 6.1	141.9 ± 9.3	0.0002
LVEF n. Simpson–%, *n* = 8	61.1 ± 2.2	67.1 ± 2.9	n.s. (0.0793)

*Values are presented as mean ± SEM. A Mann-Whitney U test was applied. P < 0.05 was considered significant. NTproBNP was measured in the superior vena cava (SVC) of all subjects. BSA, body surface area; D, diastolic; dPAP, diastolic pulmonary arterial pressure; dTPG, diastolic transpulmonary pressure gradient; HR, higher risk; HPAH, heritable pulmonary arterial hypertension; IPAH, idiopathic pulmonary arterial hypertension; LR, lower risk; LV, left ventricle; LVEF, left ventricular ejection fraction, LVOTO, left ventricular outflow tract obstruction; mPAP, mean pulmonary arterial pressure; mRAP, mean right atrial pressure; mSAP, mean systemic arterial pressure; mTPG, mean transpulmonary pressure gradient; NT-proBNP, N-terminal pro b-type natriuretic peptide; PAAT, pulmonary artery acceleration time; PAWP, pulmonary arterial wedge pressure; PAH, pulmonary arterial hypertension; PH, pulmonary hypertension; PSAX, parasternal short axis; PVR, pulmonary vascular resistance; PVRi, pulmonary vascular resistance index; Qpi, pulmonary flow index; Qsi, systemic flow index; RV, right ventricle; RVAWD, right ventricular anterior wall diameter; RVEDD, right ventricular end-diastolic diameter; S, systolic; sPAP, systolic pulmonary arterial pressure; SVR, systemic vascular resistance; TAPSE, tricuspid annular plane systolic excursion; TRV, tricuspid regurgitation velocity; WHO, World Health Organization*.

### Biomarker Assays

EDTA whole blood samples obtained during cardiac catheterization were immediately centrifuged for 10 min at 1300g. Aldosterone plasma concentrations were measured using the LIAISON Aldosterone Assay (REF 310450, DiaSorin Inc., Stillwater, MN, USA), a chemiluminescent immunoassay (CLIA) in routine clinical use. One sample had to be diluted 1:10 because the initial measured aldosterone concentration was above the detection limit of 1000 pg/ml. IL-7, VEGF-C, ICAM-1 and VCAM-1 plasma concentrations were determined using Meso Scale Discovery's Multi Array technology (MSD, Rockville, MD, USA) according to the manufacturer's instructions. IL-7 (dilution 1:2) plasma concentrations were determined within the Cytokine Panel, VEGF-C (dilution 1:2) within the Angiogenesis Panel and both ICAM-1 and VCAM-1 (dilution 1:1000) within the Vascular Injury Panel. Briefly, the 96-well plate for the Angiogenesis Panel was blocked with 150 μL/well of Blocker A solution and incubated at room temperature with gentle shaking for 1 hour. After blocking, the Angiogenesis Panel follows the same instructions as the Vascular Injury Panel and the Cytokine Panel. All plates were washed with 150 μL/well of wash buffer and 50 μL (Cytokine Panel and Angiogenesis Panel)/25 μL (Vascular Injury Panel) of diluted samples, calibrators or controls were added per well and incubated at 4°C overnight. The next day, the plates were washed three times with wash buffer and 25 μL of detection antibody was added to each well and incubated with gentle shaking for 2 h (Cytokine Panel and Angiogenesis Panel) / for 1 h (Vascular Injury Panel) at room temperature. After the incubation, the plate was again washed three times and 150 μL of Read Buffer was added per well. Signal intensities were determined with the MESO QuickPlex SQ 120 instrument (MSD, Rockville, MD, USA) and analysis was performed using the Discovery Workbench software 4.0 (MSD, Rockville, MD, USA), according to the manufacturer's instructions.

### Statistical Analysis

Statistical analyses were performed with the GraphPad Prism 6 software and R. Data are expressed as mean ± standard error (SEM) and were tested for normal distribution with D'Agostino-Pearson omnibus, Shapiro-Wilk, and Kolmogorov-Smirnov tests. If the data passed all three normality tests, unpaired *t*-test with Welch's correction was performed. If the data were not normally distributed, Mann-Whitney U was used for group comparisons. *P*-values < 0.05 were considered significant. For the gradient analysis, we used Wald χ^2^ test implemented using mixed effects models with log2 of fold change (FC) between two catheterization sites (AAO vs. PA for the transpulmonary gradient) as the dependent variable, groups (PAH or control) as an independent variable, and each patient as a random effect [log2(FC)~Group, random = ~1|Patient]. Outliers were removed using the Hampel filter. To remove the most improbable values, we set the parameter sigma to 10. Samples with an outlier in at least one of the three sites were removed. For correlation analysis, normal distribution was tested with Shapiro-Wilk test and either Pearson or Spearman correlation test was used.

## Results

### Compartment-Specific Plasma Concentrations of IL-7, VEGF-C and Aldosterone in Pediatric PH Patients vs. Non-PH Controls

**Figure 1:**
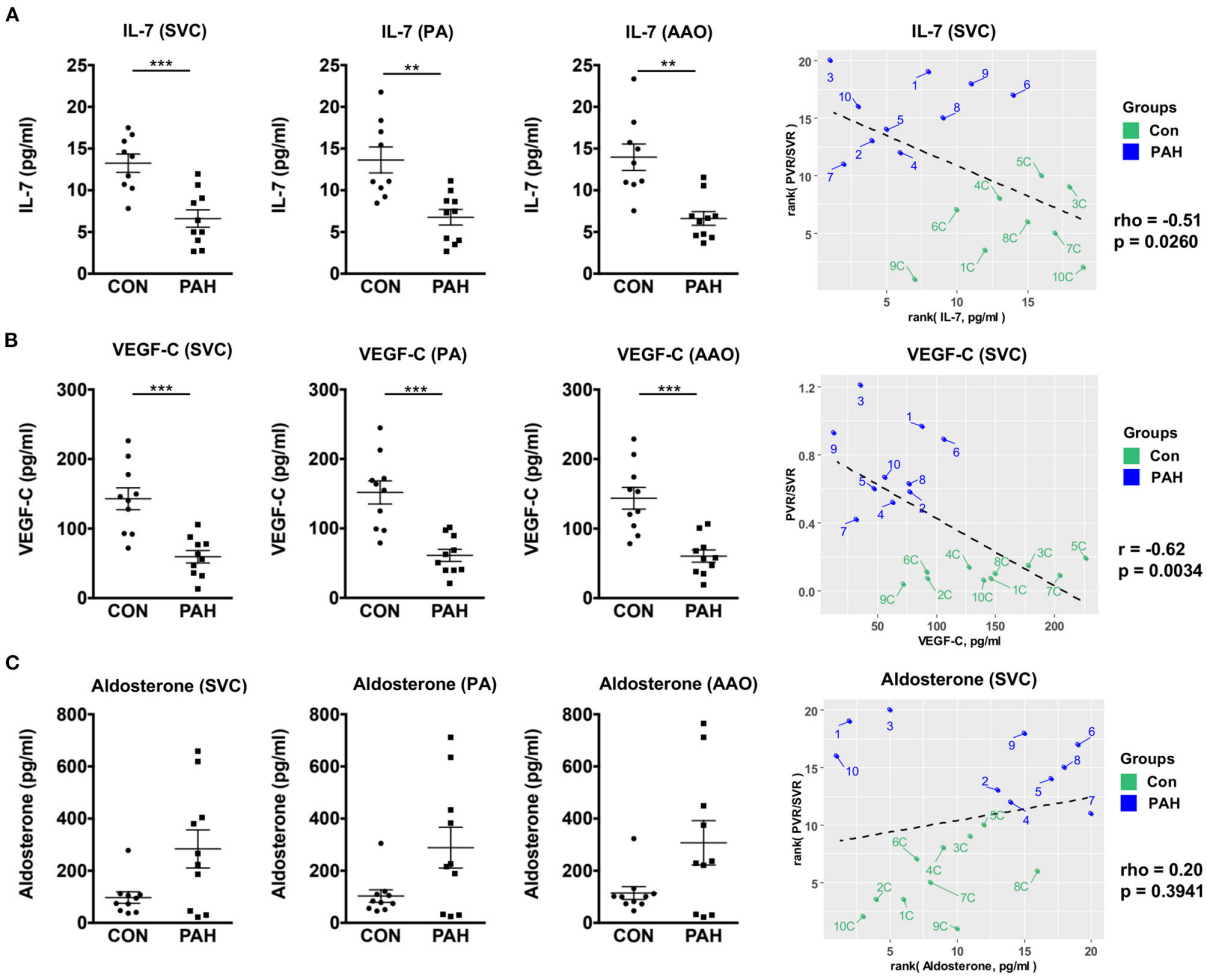
Compartment-specific plasma concentrations of IL-7, VEGF-C and aldosterone in pediatric PH patients vs. non-PH controls. For group comparisons **(A–C)** mean ± SEM are shown. Statistical test: Unpaired *t*-test with Welch's correction or Mann-Whitney U test. ^**^p < 0.01, ^***^p < 0.001. **(A)** Plasma levels of IL-7 were significantly downregulated in PH in all three sites (SVC, PA, and AAO). **(B)** Plasma levels of VEGF-C were also significantly downregulated in PAH in all three sites. IL-7 **(A)** and VEGF-C **(B)** had moderate (negative) correlation with hemodynamic variables (PVR/SVR ratio). **(C)** No significant differences in plasma aldosterone concentrations were detected, however, the concentrations tended to be higher in PAH in all three sites. Aldosterone only weakly correlated with the measured hemodynamics (PVR/SVR ratio). For correlation analysis, we used either Spearman's rho **(A,C)** or Pearson's r **(B)**, as appropriate. AAO, ascending aorta; IL-7, interleukin-7; PA, pulmonary artery; PH, pulmonary hypertension; PVR, pulmonary vascular resistance; SVC, superior vena cava; SVR, systemic vascular resistance; VEGF-C, vascular endothelial growth factor C.

First, we determined the plasma concentrations of immune-modulatory IL-7: Children with PAH had approximately 50% lower IL-7 concentrations in the SVC (6.61 ± 1.04 vs. 13.25 ± 1.10 pg/mL, *p* < 0.001), PA (6.77 ± 0.93 vs. 13.63 ± 1.55 pg/mL, *p* < 0.01) and AAO (6.62 ± 0.82 vs. 13.97 ± 1.58 pg/mL, *p* < 0.01) vs. in the corresponding sites of controls (Figure 1A). The SVC IL-7 concentrations correlated negatively with the pulmonary vascular resistance (PVR)/systemic vascular resistance (SVR) ratio, an accepted indicator of PAH severity (rho = −0.51, *p* = 0.026; Figure 1A, right panel). In contrast to our two previous studies on miRNA (11) and lipid metabolites (12), we did not find any trans-RV (PA vs. SVC) or transpulmonary (AAO vs. PA) IL-7 gradients (data not shown).

**Figure 2:**
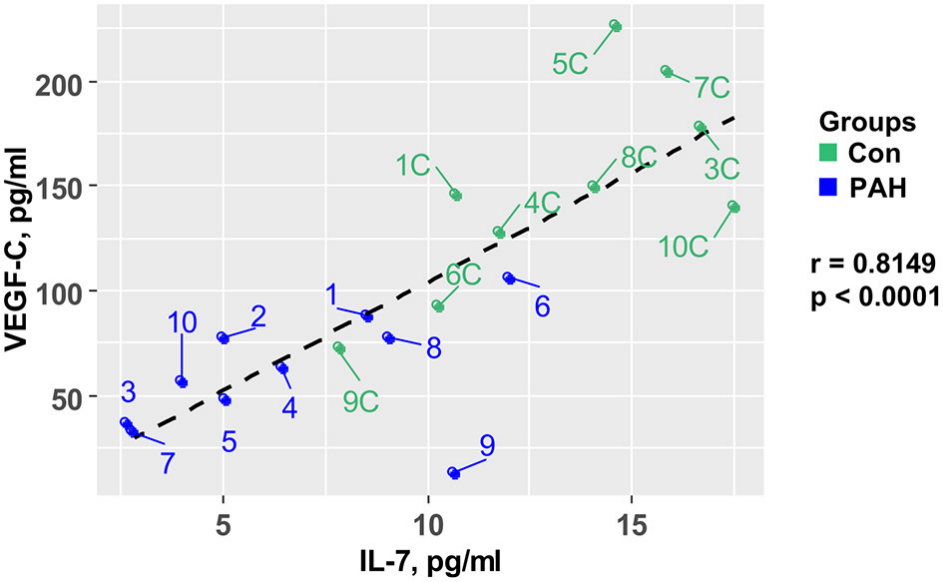
Central-venous IL-7 in pediatric samples strongly positively correlates with VEGF-C. IL-7 plasma concentrations correlate with VEGF-C plasma concentrations measured in the superior vena cava (SVC) of 10 PH patients and 10 non-PH controls. Statistical test: Pearson. IL-7, interleukin-7; PH, pulmonary hypertension; VEGF-C, vascular endothelial growth factor C.

In order to test whether pro-angiogenic factors are decreased in pediatric PAH, we measured plasma VEGF-C concentrations, and found – similarly to IL-7–approximately 59% lower circulating VEGF-C levels in pediatric PAH vs. controls (SVC, PA, AAO; *p* < 0.001) that correlated negatively with the PVR/SVR ratio (*r* = −0.62, *p* = 0.0034; Figure 1B). Central-venous IL-7 strongly positively correlated with VEGF-C (*r* = 0.81, *p* < 0.0001; Figure 2), suggesting a possible shared upstream pathway negatively affecting IL-7 mediated immunomodulation and VEGF-C induced angiogenesis in pediatric PAH. In addition, there was a trend toward higher aldosterone levels in PAH vs. controls in children, but there was no correlation between plasma aldosterone and PAH severity (PVR/SVR ratio; Figure 1C right panel). We had previously studied 41 adult patients with IPAH, and 8 age-matched, unrelated controls (16). Aldosterone plasma concentrations were similar in the PAH children under study (SVC, mean 283.6 ± 73.0 pg/ml; Figure 1C) and those of adults with IPAH (mean 248.5 ± 38.8 pg/ml) (16).

### Transpulmonary Decrease in the Vascular Injury Markers ICAM-1 and VCAM-1 in Pediatric PH Patients vs. Non-PH Controls

**Figure 3:**
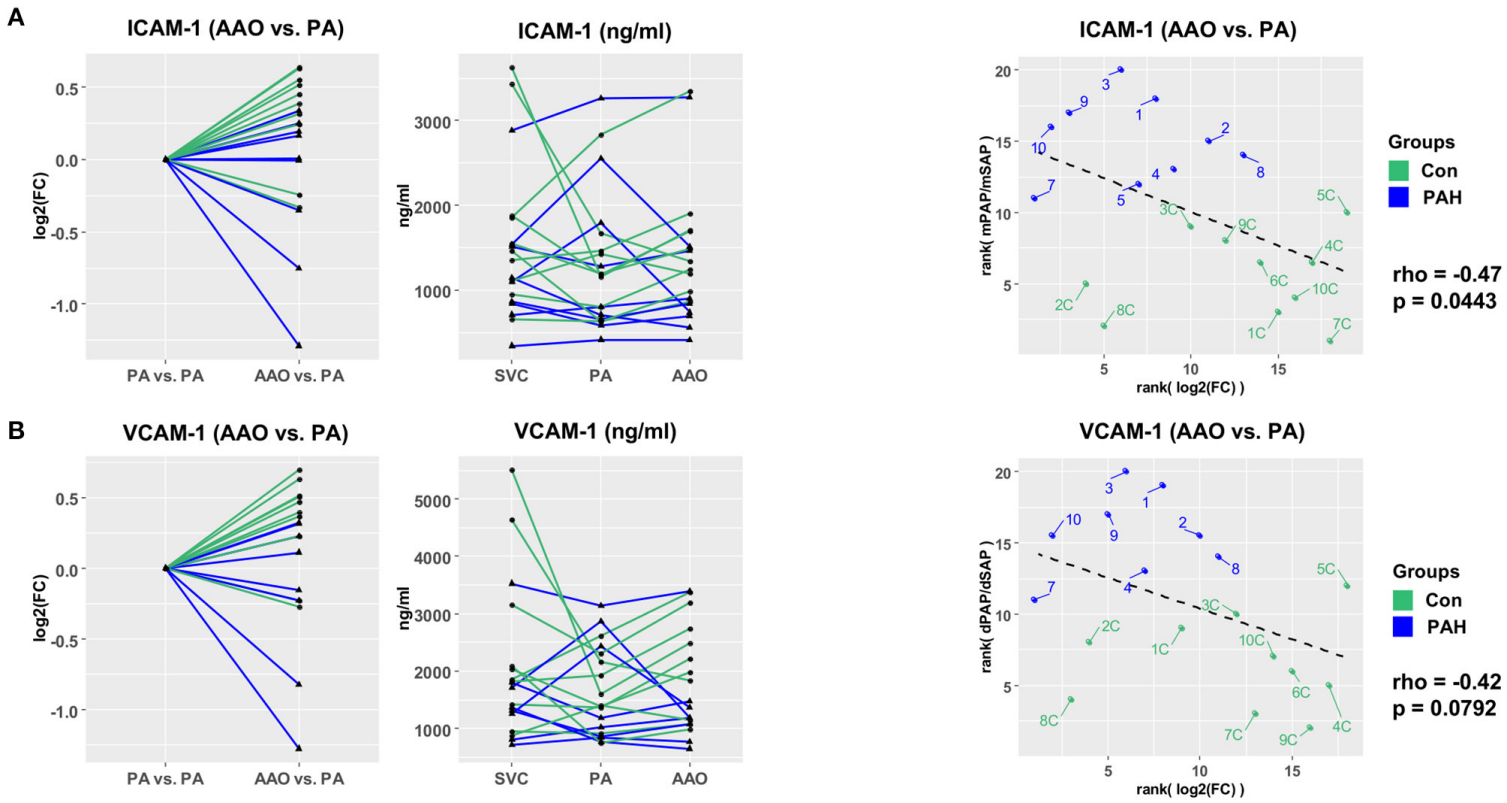
Vascular injury markers ICAM-1 and VCAM-1 have differential transpulmonary gradients in pediatric PH vs. non-PH controls. **(A)** Step-down in PAH (FC = 0.86) and step-up in controls (FC = 1.21) for ICAM-1 levels. **(B)** Step-down in PAH (FC = 0.85) and step-up in controls (FC = 1.28) for VCAM-1. For transpulmonary gradient analysis, we used Wald χ^2^ test (linear mixed effects models). For correlation analysis, we used Spearman's rho **(A,B)**. ICAM-1 and VCAM-1 had moderate negative correlation with hemodynamic variables (mPAP/mSAP; dPAP/dSAP). AAO, ascending aorta; dPAP, diastolic pulmonary arterial pressure; dSAP, diastolic systemic arterial pressure; ICAM-1, intercellular adhesion molecule 1; mPAP, mean pulmonary arterial pressure; mSAP, mean systemic arterial pressure; PA, pulmonary artery; PH, pulmonary hypertension; SVC, superior vena cava; VCAM-1, vascular cell adhesion molecule 1.

Next, we investigated the vascular injury markers ICAM-1 and VCAM-1 in pediatric PH. Most patients had a step down in ICAM-1 (Figure 3A) and VCAM-1 (Figure 3B) plasma concentrations across the pulmonary circulation (AAO vs. PA gradients) with the following fold changes (FC): FC_PAH_ICAM−1_ = 0.86, FC_Control_ICAM−1_ = 1.21, FC_PAH_VCAM−1_ = 0.85, and FC_Control_ICAM−1_ = 1.28. Both ICAM-1 and VCAM-1 transpulmonary gradients had moderate negative correlation with surrogates of PAH severity: ICAM-1 (AAO vs. PA) vs. mPAP/mSAP ratio (rho = −0.47, *p* = 0.044; Figure 3A) and VCAM-1 (AAO vs. PA) vs. dPAP/dSAP ratio (rho = −0.42, *p* = 0.079; Figure 3B). The decrease in transpulmonary ICAM-1 and VCAM-1 concentrations in PAH children (fold change 0.86 and 0.85, respectively; Figures 3A,B) indicates either intrapulmonary uptake or increased intrapulmonary degradation of these vascular injury mediators that drive endothelial dysfunction (1, 2).

## Discussion

Here, we report for the first time invasive, compartment-specific levels of circulating IL-7, VEGF-C, aldosterone, ICAM-1 and VCAM-1 in children with moderate PH. We found a very pronounced decrease in plasma IL-7 concentrations in pediatric PH patients vs. non-PH controls, which inversely correlated with precapillary PH severity, as assessed by the ratio of PVR/SVR. Similarly to IL-7, we found approximately 59% lower circulating VEGF-C levels in pediatric PAH vs. controls that correlated negatively with the PVR/SVR ratio. Central-venous IL-7 strongly positively correlated with VEGF-C. Additionally, there was a trend toward higher aldosterone levels in pediatric PH patients vs. controls, with levels similar to those previously measured in adults (16). Transpulmonary gradient analysis revealed that most patients had a step down in ICAM-1 and VCAM-1 plasma concentrations across the pulmonary circulation, indicating either intrapulmonary uptake or increased intrapulmonary degradation of these vascular injury markers.

The rationale to investigate circulating IL-7 in (pediatric) PAH was its aforementioned role in NK and Treg cell survival (4–7), both of which are thought to be crucial for the adaptations necessary in PAH and RVD. NK cells are the innate immune equivalent of cytotoxic T cells that spot stressed or damaged cells and induce targeted cell death. NK cells are mainly circulating in the blood stream, but resident populations have also been found in lung, liver, and lymph nodes. NK cells are reduced in number and function in peripheral blood of adults with PAH, compared to control subjects, releasing less cytotoxic granules (4). Dysregulated immunity resulting from deficient Treg activity in athymic rats contributes to increased inflammation, leading to macrophage activation and rapid progressive PAH and RV failure (7).

Consistent with our finding of the greatly decreased IL-7 concentrations in the systemic and pulmonary circulation of children with PAH (Figure 1), others had demonstrated reduced expression of IL-7 receptor (IL-7R) in circulating PBMC and CD4+ T-cells from adult scleroderma (SSc) patients with PAH vs. those without PAH (17).

According to the aforementioned SSc-PAH study and our current findings, we postulate that blood cell IL-7/IL7R signaling is decreased in adult and pediatric PAH. However, increased immunoreactivity for IL-7 was found in tertiary (ectopic) lymphoid tissues, i.e., highly organized perivascular follicles, in adult idiopathic PAH (IPAH) lungs, suggesting specific immune-adaptive mechanisms in the pathophysiology of the disease, at least in adults (18). So far, it is unclear whether intrapulmonary IL-7 signaling is a detrimental driver or counterregulatory protector in pulmonary lymphoid neogenesis (18), and other hallmarks of PAH pathobiology.

Additionally, we revealed for the first time decreased levels of VEGF-C in pediatric PAH patients. VEGF-C primarily signals through vascular endothelial growth factor receptor 3 (VEGFR3) which is expressed in endothelial cells and regulates cardiovascular development and lymphangiogenesis (19, 20). VEGFR3 has been found to be a positive regulator of bone morphogenetic protein receptor 2 (BMPR2) signaling in PAH and knockout of VEGFR3 in endothelial cells caused exacerbation of hypoxia-induced PH in mice (21). Consistent with our finding of significantly decreased VEGF-C plasma levels in PH children, the expression of VEGFR3 (both VEGFR3 mRNA and protein) was found to be decreased in pulmonary arterial endothelial cells isolated from adult PAH patients vs. controls (21).

In our previous human biomarker study, ICAM-1 was increased in adult IPAH and CTD-PAH vs. controls, whereas VCAM-1 and pro-inflammatory, anti-angiogenic interleukin 12 (IL-12) were significantly elevated in CTD-PAH only (16). In the same study, we showed for the first time that a clinically relevant aldosterone/galectin-3 axis exists in adult PAH and associates with clinical symptoms (WHO functional class) (16).

## Conclusion

The limitations of our study are common in biomarker studies on a rare disease, and include small sample size (children), non-healthy controls (invasive diagnostics), and a wide age range. Nevertheless, this article is the first report on decreased circulating IL-7 and VEGF-C plasma concentrations in human PAH and their inverse correlations with invasive surrogates of PAH severity. Additional and larger studies are warranted to explore the role of the immune-modulatory IL-7 and VEGF-C at multiple locations, cells and tissues (circulation, pulmonary vascular/lymph vessels, RV myocardium) in pediatric and adult PAH.

## Data Availability Statement

The raw data supporting the conclusions of this article will be made available by the authors, without undue reservation.

## Ethics Statement

The studies involving human participants were reviewed and approved by Hannover Medical School (#2200). Written informed consent to participate in this study was provided by the participants' legal guardian/next of kin.

## Author Contributions

FD provided clinical data, performed experiments and statistical analysis, produced display items, and wrote parts of the manuscript. EL supervised, performed experiments and statistical analysis, and produced display items. PC performed advanced data analysis and produced display items. HB, CH, and GH collected blood samples during pediatric cardiac catheterizations. GH conceptualized, designed and supervised the study, performed experiments, and wrote the manuscript. All authors reviewed and revised the manuscript for important intellectual content.

## Funding

This study was supported by the German Research Foundation (DFG; HA4348/2-2 and KFO311 HA4348/6-2 to GH) and the European Pediatric Pulmonary Vascular Disease Network (www.pvdnetwork.org). GH receives additional funding from the Federal Ministry of Education and Research (BMBF ViP+ program 03VP08053; BMBF 01KC2001B).

## Conflict of Interest

The authors declare that the research was conducted in the absence of any commercial or financial relationships that could be construed as a potential conflict of interest.

## Publisher's Note

All claims expressed in this article are solely those of the authors and do not necessarily represent those of their affiliated organizations, or those of the publisher, the editors and the reviewers. Any product that may be evaluated in this article, or claim that may be made by its manufacturer, is not guaranteed or endorsed by the publisher.

## Abbreviations

AAO: ascending aorta
BSA: body surface area
BMPR2: bone morphogenetic protein receptor 2
dPAP: diastolic pulmonary arterial pressure
dTPG: diastolic transpulmonary pressure gradient
dSAP: diastolic systemic arterial pressure
HGF: hepatocyte growth factor
HPAH: heritable pulmonary arterial hypertension
ICAM-1: intercellular adhesion molecule 1
IL-7: interleukin-7
IPAH: idiopathic pulmonary arterial hypertension
LV: left ventricle
LVEDP: left ventricular end-diastolic pressure
LVEF: left ventricular ejection fraction
LVOTO: left ventricular outflow tract obstruction
mPAP: mean pulmonary arterial pressure
mRAP: mean right atrial pressure
mSAP: mean systemic arterial pressure
mTPG: mean transpulmonary pressure gradient
NK cells: natural killer cells
NTproBNP: N-terminal pro b-type natriuretic peptide
PA: pulmonary artery
PAAT: pulmonary artery acceleration time
PAWP: pulmonary arterial wedge pressure
PAH: pulmonary arterial hypertension
PH: pulmonary hypertension
PSAX: parasternal short axis
PVRi: pulmonary vascular resistance index (indexed to body surface area)
Qpi: pulmonary flow index
Qsi: systemic flow index
RV: right ventricle
RVAWD: right ventricular anterior wall diameter
RVEDD: right ventricular end-diastolic diameter
sPAP: systolic pulmonary arterial pressure
sSAP: systolic systemic arterial pressure
SVC: superior vena cava
SVR: systemic vascular resistance
TAPSE: tricuspid annular plane systolic excursion
VEGF-C: vascular endothelial growth factor C
VEGFR3: vascular endothelial growth factor receptor 3
VCAM-1: vascular cell adhesion molecule 1
WHO: world health organization.
